# A Comparative Analysis of the Murine Thymic Microenvironment in Normal, Autoimmune, and Immunodeficiency States

**DOI:** 10.1155/1997/69270

**Published:** 1997

**Authors:** Yuichi Takeoka, Shao-Yuan Chen, Richard L. Boyd, Koichi Tsuneyama, Nobuhisa Taguchi, Shinji Morita, Hisashi Yago, Seishi Suehiro, Aftab A. Ansari, Leonard D. Shultz, M. Eric Gershwin

**Affiliations:** 1 Division of Rheumatology Allergy and Clinical Immunology University of California at Davis School of Medicine Davis California 95616 USA; 2 Institute of Bio-Active Science Nippon Zoki Pharmaceutical Co. Ltd. Yashiro Hyogo 673-14 Japan; 3 Department of Pathology and Immunology Monash University Prahran Victoria 3181 Australia; 4 Department of Pathology Emory University School of Medicine Atlanta Georgia 30322 USA; 5 Jackson Laboratory Bar Harbor Maine 04609 USA; 6 Department of Pathology Toyama City Hospital Imaizumi Toyama 939 Japan

**Keywords:** Thymic microenvironment, NZB mouse, SM/J mouse, NOD mouse, NOD-scib mouse, Aly/aly mouse, Motheaten (Hpch^me/Hpch^me) mouse

## Abstract

It is widely accepted that the thymic microenvironment regulates normal thymopoiesis through a highly coordinated and complex series of cellular and cytokine interactions. A direct corollary of this is that abnormalities within the microenvironment could be of etiologic significance in T-cell-based diseases. Our laboratory has developed a large panel of monoclonal antibodies (mAbs) that react specifically with epithelial or nonepithelial markers in the thymus. We have taken advantage of these reagents to characterize the thymic microenvironment of several genetic strains of mice, including BALB/cJ, C57BL/6J, NZB/BlnJ, SM/J, NOD/Ltz, NOD/Ltz-*scid/sz*, C57BL/6J-*Hcph* m^e^/*Hcph* m^e^, and ALY/NscJcl-*aly/aly* mice, and littermate control animals. We report herein that control mice, including strains of several backgrounds, have a very consistent phenotypic profile with this panel of monoclonal antibodies, including reactivity with thymic epithelial cells in the cortex, the medulla and the corticomedullary junction, and the extracellular matrix. In contrast, the disease-prone strains studied have unique, abnormal staining of thymic cortex and medulla at both the structural and cellular levels. These phenotypic data suggest that abnormalities in interactions between developing thymocytes and stromal cells characterize disease-prone mice.


*Developmental Immunology*, 1997, Vol. 5, pp. 79–89
Reprints available directly from the publisher
Photocopying permitted by license only

© 1997 OPA (Overseas Publishers Association)
Amsterdam B.V. Published under license in The Netherlands
under the Harwood Academic Publishers imprint
part of The Gordon and Breach Publishing Group
Printed in Malaysia

### A Comparative Analysis of the Murine Thymic Microenvironment in Normal, Autoimmune, and Immunodeficiency States

YUICHI TAKEOKA[a,b], SHAO-YUAN CHEN[a], RICHARD L. BOYD[c], KOICHI TSUNEYAMA[a,†], NOBUHISA TAGUCHI[b], SHINJI MORITA[b], HISASHI YAGO[b], SEISHI SUEHIRO[b], AFTAB A. ANSARI[d], LEONARD D. SHULTZ[e] and M. ERIC GERSHWIN[a,*]

[a]*Division of Rheumatology, Allergy and Clinical Immunology, University of California at Davis, School of Medicine, Davis, California 95616* [b]*Institute of Bio-Active Science, Nippon Zoki Pharmaceutical Co. Ltd., Yashiro, Hyogo 673-14, Japan* [c]*Department of Pathology and Immunology, Monash University, Prahran, Victoria, 3181, Australia* [d]*Department of Pathology, Emory University, School of Medicine, Atlanta, Georgia 30322* [e]*Jackson Laboratory, Bar Harbor, Maine 04609*

*(Received 14 October 1996; In final form 10 December 1996)*

It is widely accepted that the thymic microenvironment regulates normal thymopoiesis through a highly coordinated and complex series of cellular and cytokine interactions. A direct corollary of this is that abnormalities within the microenvironment could be of etiologic significance in T-cell-based diseases. Our laboratory has developed a large panel of monoclonal antibodies (mAbs) that react specifically with epithelial or nonepithelial markers in the thymus. We have taken advantage of these reagents to characterize the thymic microenvironment of several genetic strains of mice, including BALB/cJ, C57BL/6J, NZB/BlnJ, SM/J, NOD/Ltz, NOD/Ltz-*scid/sz*, C57BL/6J-*Hcph$^{me}$/Hcph$^{me}$*, and ALY/NscJcl-*aly/aly* mice, and littermate control animals. We report herein that control mice, including strains of several backgrounds, have a very consistent phenotypic profile with this panel of monoclonal antibodies, including reactivity with thymic epithelial cells in the cortex, the medulla and the corticomedullary junction, and the extracellular matrix. In contrast, the disease-prone strains studied have unique, abnormal staining of thymic cortex and medulla at both the structural and cellular levels. These phenotypic data suggest that abnormalities in interactions between developing thymocytes and stromal cells characterize disease-prone mice.

*Keywords:* Thymic microenvironment, NZB mouse, SM/J mouse, NOD mouse, NOD-*scid* mouse, *Aly/aly* mouse, Motheaten (*Hpch$^{me}$/Hpch$^{me}$*) mouse

### INTRODUCTION

The unique capacity of the thymus for rapidly generating functionally mature, self-MHC-restricted yet self-tolerant T cells is encompassed within its stromal microenvironment, the specialized nature of which derives from both the diverse cellular content and the complex structural organization (Boyd et al., 1993). Recently, interest in the nature and function of the thymic microenvironment has intensified with the real-

*Corresponding author.
†Present address: Department of Pathology, Toyama City Hospital, Imaizumi, Toyama 939, Japan.

ization that defects in it may underlie abnormal T-cell development and hence diseases such as autoimmunity and immunodeficiency. The most important component of the microenvironment is the epithelial cells that define three major areas: the subcapsule, cortex, and medulla. Epithelial cells in the subcapsular region do not express MHC class II, but are believed to initiate thymopoiesis (Van de Wijngert et al., 1983; Boyd et al., 1992, 1993). Thymocytes presumably then contact underlying cortical epithelial cells including thymic nurse cells that express both MHC class I and II molecules and most likely have an important function in positive selection (Berg et al., 1989; Ashton-Rickardt et al., 1993). Phenotypically mature single-positive T cells ($CD4^+$ $CD8^-$ or $CD4^-$ $CD8^+$) are concentrated in the medulla, suggesting that this region completes thymocyte-positive selection (Ritter and Boyd, 1993); however, it may have been initiated earlier but was simply manifested in the medulla. In fact, medullary thymic epithelial cells induce tolerance toward class I-restricted self-peptides presented on their own surface (Oukka et al., 1996).

Clearly, the thymic microenvironment, in particular epithelial cells, are indispensable for T-cell maturation. The logical corollary to this is that defects in the cellular content or spatial organization of the thymic stroma may be of etiologic significance in some diseases. In this context, we previously described thymic stromal abnormalities in several murine models of human systemic lupus erythematosus (SLE), including New Zealand Black (NZB), MRL/MP-$Fas^{lpr}$, C3H/HeJ-$Fas^{gld}$, and BXSB/MpJ.*Yaa* mice (Watanabe et al., 1993; Takeoka et al., 1995a, 1995b). In addition, there are several other genetically determined murine models for immunodeficiency and autoimmune diseases. These include SM/J, NOD, ALY/Nsc Jcl-*aly*/*aly*, and motheaten ($Hpch^{me}/Hpch^{me}$) mice. SM/J mice have high levels of natural thymocytotoxic autoantibodies (NTA) found commonly in murine lupus strains (Mittal et al., 1970; Terasaki et al., 1970; Shirai and Mellors, 1971; Huang et al., 1973; Goldblum et al., 1975; Eisenberg et al., 1979) yet surprisingly do not manifest severe pathogenic changes. NOD mice are a well-known model of human insulin-dependent diabetes mellitus (IDDM), an autoimmune disease targeting the insulin-secreting cells in pancreatic islets (Tochino, 1987). The "motheaten" autosomal recessive mutation disrupts the structural gene for a cytoplasmic protein tyrosine phosphatase called hematopoetic cell phosphatase (Shultz et al., 1993). Mice homozygous for motheaten ($Hpch^{me}/Hpch^{me}$) manifest immunodeficiency and autoimmunity and exhibit severe defects in the development and function of both T and B lymphocytes Green and Shultz, 1975). Finally, the "alymphoplasia" mouse-termed ALY/Nsc Jcl-*aly*/*aly* from the gene symbol "*aly*" is a spontaneous autosomal recessive mutant that causes a systemic absence of lymph nodes and Peyer's patches, and reduced levels of IgM and severely depressed levels of IgG and IgA (Miyawaki et al., 1994). Because we have shown in earlier studies that lupus-prone mice have characteristic defects in the thymic microenvironment (Watanabe et al., 1993; Takeoka et al., 1995a, 1995b), the present study was undertaken to determine whether such abnormalities are generic to T-cell disorders in general.

### RESULTS

### Classification of the mAbs

Fourteen mAbs used in this study have been classed into three categories, based on their reactivity pattern with normal thymic tissue: (1) those reactive with thymic epithelial cells, (2) those reactive with both thymic epithelial cells and thymic lymphoid cells (thymocytes), and (3) those reactive with nonepithelial stromal cells. Six of these mAbs detect thymic epithelial cells including pan epithelium (MTS1 and MTS5), subcapsular and medullary epithelium (MTS10), cortical epithelium (MTS44), and medullary epithelial-cell clusters (MTS20 and MTS24). MTS20 and MTS24 recognize the isolated clusters of medulla thymic epithelium and are pan-epithelial markers early in thymic ontogeny (Godfrey et al., 1990, Blackburn et al., 1996). Three of these mAbs (MTS33, MTS35, and MTS37) stain both epithelial and nonepithelial cells. MTS33 recognizes ThB (Boyd et al., 1993), MTS35 recognizes thymic shared Ag-1 (TSA-1) (Godfrey et al., 1992; Boyd et al., 1993), and MTS37 recognizes heat-stable antigen (HSA) (Boyd et al., 1993) on thymocytes. MTS12 recognizes vascular endothelium, MTS16 recognizes collagenlike extracellular matrix, and MTS28 recognizes macrophage- (Mϕ-) like cells. Rabbit anti-keratin antibody (Charles River Pharmservices, Southbridge, MA) was used to detect all epithelial cells in thymus. Finally, a rat anti-mouse Mac-1 antibody was used to detect Mac-1 antigen (CD11b/CD18 as $\alpha_M\beta_2$ integrin) on macrophage, granulocytes, natural killer cells, and B-1 cells. The nomenclature, classification of the clusters of thymic epithelial staining (CTES) (Kampinga et al., 1989), and the characteristics of each of these mAb are summarized in Table I, and the detailed specificities of these mAbs in thymus are illustrated in Fig. 1.

### Thymic Architecture of Control Mice: C57BL/6, BALB/c, + /*Hcph*$^{me}$-Heterozygous, and + /*aly*-heterozygous mice

MTS1 and MTS5 are essentially pan-epithelium reagents, similar to anti-keratin, although there are minor differences. MTS44 stains the entire cortical epithelial cell network and very infrequent isolated epithelial cells in the medulla (Fig. 2). There were a few small areas of MTS44-negative epithelial cells in the cortex. MTS10 is specific for medullary and subcapsular epithelium; these are also morphologically distinct from cortical epithelial cells being less stellate and more compact (Fig. 3). Within the medullary network, there are small clusters of epithelial cells that are stained by MTS33 (ThB), which also stains cortical but not medullary thymocytes (Godfrey et al., 1990; Boyd et al., 1993). MTS20 and MTS24 also label similar if not identical isolated clusters of medullary epithelium (Fig. 3).

MTS35, which recognizes the thymic shared antigen-1 (TSA-1) (Godfrey et al., 1990, 1992) stained thymocytes in the outer cortex in the thymus of control mice (Fig. 4). MTS37, which recognizes heat-stable antigen (HSA) (Boyd et al., 1993), stained thymocytes in both the cortical and medullary regions.

MTS16 strongly labels the extracellular matrix associated with the capsule, interlobular septa, and vasculature (Fig. 5). MTS12 (CD31) stained all vascular endothelial cells. Finally, Mac-1 and MTS28 recognized isolated macrophage- (Mϕ-) like cells, which are widely distributed in the thymus.

The thymic architecture found in + /*Hcph*$^{me}$-heterozygous and + /*aly*-heterozygous mice were indistinguishable from the normal C57BL/6 and BALB/c mice (Table II) and CBA mice (Godfrey et al., 1990).

TABLE I Classification of mAb to Thymic Microenvironmental Elements[a]

| Predominant staining | mAb | CTES[b] classification | Thymic epithelium: Subcapsule | Cortex | Medulla | Remarks |
|---|---|---|---|---|---|---|
| Thymic epithelium | MTS1 | I | ++ | ++ | ++ | |
| | MTS5 | I | ++ | ++ | + | |
| | MTS10 | II | ++ | − | ++ | |
| | MTS44 | III.B | ± | ++ | ± | |
| | MTS20 | IV.A | − | − | ± | Isolated cluster of thymic epithelium |
| | MTS24 | IV.A | − | − | ± | Isolated cluster of thymic epithelium |
| | anti-keratin | pan epithelium | ++ | ++ | ++ | |
| Thymic epithelium and nonepithelial thymic cells | MTS37 | XX.c | − | ± | ± | HSA of thymocytes (cortex, medulla) |
| | MTS33 | XX.d | [c] | [c] | ± | ThB of thymocytes (cortex) |
| | MTS35 | XX.d | [c] | [c] | ± | TSA-1 of thymocytes (outer cortex) |
| Nonepithelial thymic stromal cells | MTS12 | N/D[d] | − | − | − | Vascular endothelium |
| | MTS16 | N/D[d] | − | − | − | Extracelullar matrix |
| | Mac-1 | N/D[d] | − | − | − | Isolated Mϕ-like cells |
| | MTS28 | N/D[d] | − | − | − | Isolated Mϕ-like cells |

[a]The specificities of MTS mAb on control mouse thymus sections. − : 0%; ±: <25%; + : 25 to 75%; + + : >75% of thymic epithelial cells are stained.
[b]CTES: clusters of thymic epithelial staining, classification as defined by Kampinga et al., 1989.
[c]Degree of stromal cell staining is masked by thymocytes.
[d]N/D: not defined.

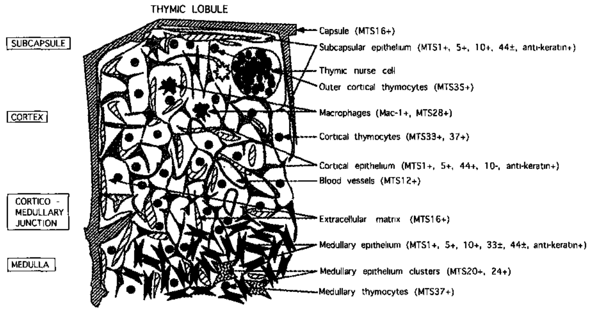

FIGURE 1 The thymic reactivity of the panel of 14 mAb used in this study was classified into three categories, based on their reactive pattern with normal thymic tissue: (1) those reactive with thymic epithelial cells (MTS1, 5, 10, 20, 24, 44, anti-keratin); (2) those reactive with both thymic epithelial cells and thymocytes (MTS33, 35, 37); and (3) those reactive with nonepithelial stromal cells (MTS12, 16, 28, Mac-1).

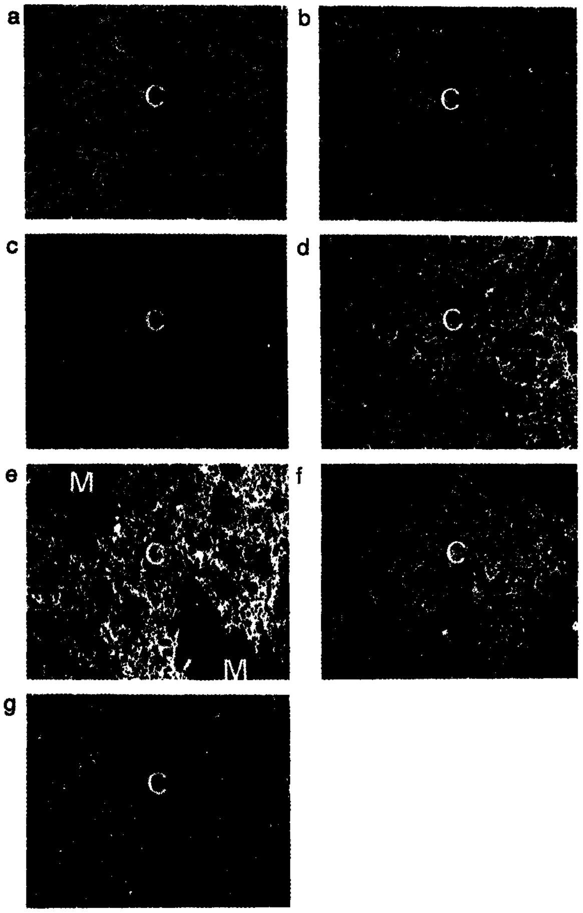

FIGURE 2 MTS44 (cortex) staining of thymus from (a) C57BL/6, (b) NZB, (c) SM/J, (d) NOD, (e) NOD-*scid*, (f) $Hpch^{me}/Hpch^{me}$, or (g) *aly/aly* mice; M: medulla, C: cortex. The cortical epithelial network was diffuse, less continuous, and irregular in (c) SM/J, (d) NOD, or (g) *aly/aly* mice. Arrows indicate diffuse cortical epithelial networks. The thymus of (e) NOD-*scid*, or (f) $Hpch^{me}/Hpch^{me}$ mice exhibit a smaller meshwork. Arrowheads indicate a smaller meshwork. The bar indicates 100 μm. ( × 200)

### Thymic Architecture of NZB Mice (Lupus)

In NZB mice, the staining pattern of the cortical epithelial network was severely disrupted and irregular using MTS1, MTS5, or MTS44 (Fig. 2). NZB mice also had large epithelial cell-free regions within the cortex that were not stained with MTS44. Surprisingly, there were clusters of MTS10-positive epithelial cells in the cortex. The medulla was also MTS10-positive as in normal mice, but the network was disrupted into smaller clusters, the staining of which was reduced for MTS1, MTS20, MTS24, and MTS33 (Fig. 3). Extracellular matrix stained with MTS16 was decreased in NZB as compared to C57BL/6 or BALB/c mice (Fig. 5). There were no differences, however, in thymocyte staining with MTS33, MTS35 (Fig. 4), or MTS37 between the thymi of NZB, and control mice. Similarly, MTS12, which recognizes vascular endothelium, and Mac-1 and MTS28, which recognize isolated macrophagelike cells, were comparable to control mice.

### Thymic Architecture of SM/J Mice (Natural Thymotoxic Antibodies)

As for NZB mice, the cortical epithelium of SM/J mice was irregular and disrupted when stained with MTS1, MTS5, or MTS44 (Fig. 2), and contained isolated clusters of MTS10-positive epithelial cells. The MTS10-positive medullary epithelial network was also disrupted and irregularly shaped (Fig. 3). However, isolated clusters stained by MTS20 (Fig. 3) or MTS24 were similar to control mice. Subcapsular epithelial cells showed a minor reduction of staining with MTS10 in the SM/J mice. SM/J mice had elevated MTS35-positive thymocytes in their corticomedullary and medullary regions (Fig. 4). However, the intensities of thymic staining with MTS33 or MTS37 were equivalent to control mice. Finally, SM/J mice had no abnormalities using MTS16 (Fig. 5), MTS12, Mac-1, or MTS28.

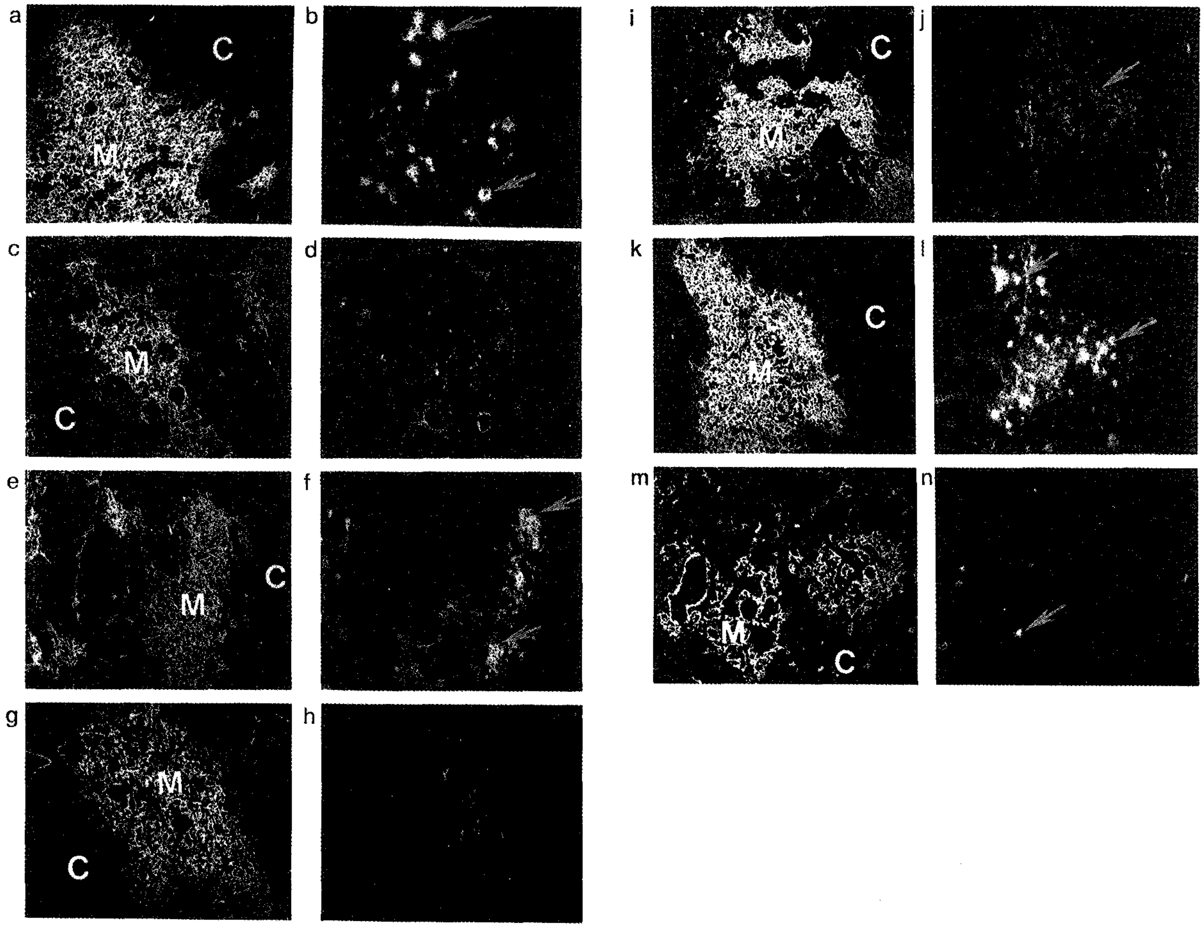

FIGURE 3 Serial sections of thymus from (a, b) C57BL/6, (c, d) NZB, (e, f) SM/J, (g, h) NOD, (i, j) NOD-*scid*, (k, l) *Hpch*$^{me}$/*Hpch*$^{me}$, or (m, n) *aly*/*aly* mice were stained with MTS10 (subcapsule/medulla; a, c, e, g, i, k, m) or MTS20 (isolated cluster of medullary epithelium; b, d, f, h, j, l, n); M: medulla, C: cortex. Thymi of (e) SM/J, (g) NOD, (i) NOD-*scid*, or (m) *aly*/*aly* mice were stained with MTS10 and exhibit diffuse atrophic clusters of medullary epithelial cells similar to (c) NZB and were contrasted with the thymus of (a) C57BL/6 mice. The clusters of irregular-shaped MTS10-positive cells were also found in the thymic cortex of (b) NZB, (e) SM/J, (g) NOD, (i) NOD-*scid*, or (m) *aly*/*aly* mice. The high-intensity clusters of MTS20-positive were reduced in (d) NZB, (h) NOD, (j) NOD-*scid*, or (n) *aly*/*aly* mice in comparison to (b) C57BL/6 mice. Arrows indicate MTS20-positive cell clusters. The bar indicates 250 μm. ( × 100)

### Thymic Architecture of NOD Mice (IDDM)

One of the major features of NOD mice was the large areas lacking epithelial cells in the cortical regions. The medullary epithelium was again irregularly shaped and disrupted (Fig. 3); however, these abnormalities were less pronounced than that of NZB mice. NOD mice had typical normal staining of cortical thymocytes by MTS33, the more outer cortical thymocytes by MTS35 (Fig. 4), and both cortical and medullary thymocytes by MTS37. The extracellular matrix stained with MTS16 was decreased in NOD mice, again similar to NZB mice (Fig. 5). Finally, staining with MTS12, Mac-1, or MTS28 was normal.

### Thymic Architecture of NOD-*scid* Mice

NOD-*scid* mice had a severe involution of the thymus, as expected, with no clearly defined cortex, only rudimentary medullary pockets and no distinct corticomedullary regions. Furthermore, both the cortical (MTS44-positive) and medullary (MTS10-positive) epithelial cells where present were irregularly shaped but

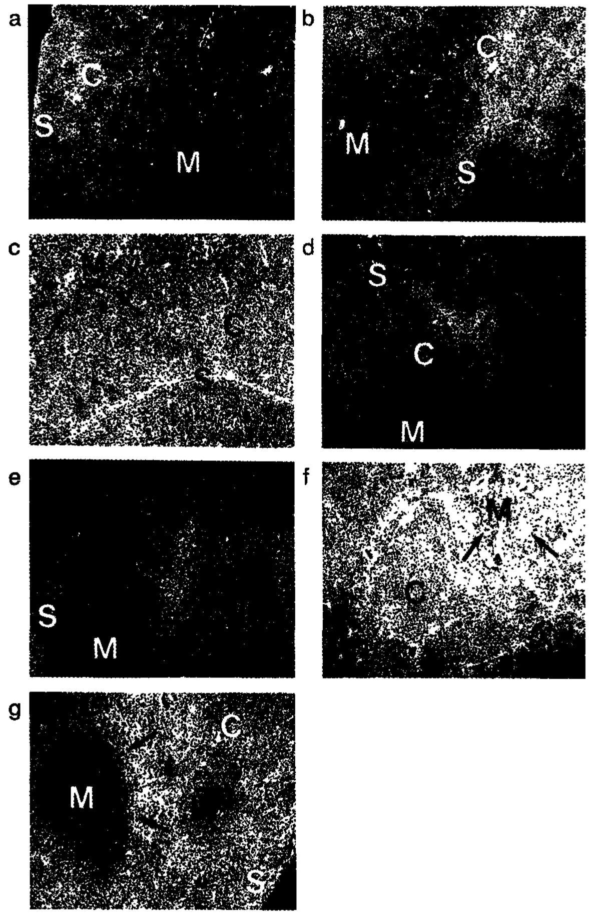

FIGURE 4 MTS35 recognizes TSA-1 present on both immature thymocytes and thymic medullary epithelial cells. MTS35 was used to stain thymus from (a) C57BL/6, (b) NZB, (c) SM/J, (d) NOD, (e) NOD-*scid*, (f) $Hpch^{me}/Hpch^{me}$, or (g) *aly/aly* mice; M: medulla, C: cortex, S: subcapsule. (a) C57BL/6 mice show normal expression. (b) NZB mice were similar to normal mice. However, (c) SM/J and (f) $Hpch^{me}/Hpch^{me}$ mice had elevated MTS35-positive thymocytes in their corticomedullary and medullary regions, and *aly/aly* mice had elevated MTS35-positive thymocytes in their corticomedullary regions. Arrows indicate MTS35-positive thymocytes in the medulla or corticomedullary regions. The bar indicates 250 µm. ( × 100)

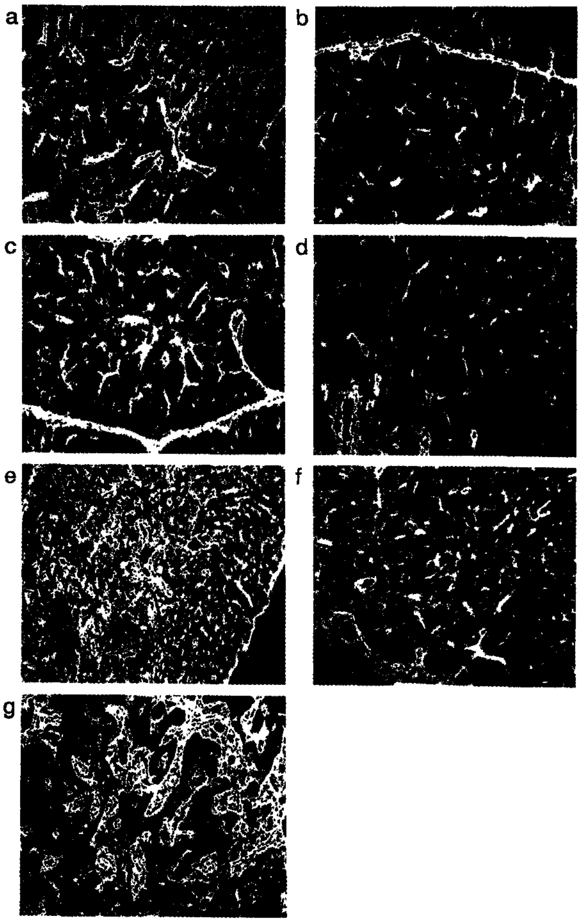

FIGURE 5 MTS16 staining of thymus from (a) C57BL/6, (b) NZB, (c) SM/J, (d) NOD, (e) NOD-*scid*, (f) $Hpch^{me}/Hpch^{me}$, or (g) *aly/aly* mice. Extracellular matrix, as recognized by MTS16, was markedly increased in (e) NOD-*scid* or (g) *aly/aly* mice. The extracellular matrix was aggregated and expressed large irregular-shaped clusters in (g) *aly/aly* mice, but (e) NOD-*scid* mice exhibited small clusters. The thymus of (b) NZB or (d) NOD mice had a mild reduction of MTS16 expression. Arrows indicate abnormalities of MTS16 staining in the thymus of *aly/aly* or NOD-*scid* mice. The bar indicates 250 µm. ( × 100)

quite different from NZB mice (Figs. 2 and 3). These staining patterns were confirmed with the pan-epithelium markers MTS1 and MTS5. Consistent with their immature status, the thymocytes were MTS33- and MTS35-positive (Fig. 4), and although very infrequent, there were isolated MTS33- and MTS35-positive epithelial cells in the medullary pockets. The density of extracellular matrix, recognized by MTS16, was increased (Fig. 5). Although less extensive because of the small size of the thymus, the staining of macrophage-like cells by Mac-1 or MTS28, or vascular endothelium by MTS12 was similar to control NOD-+ /+ , C57BL/6, or BALB/c mice.

### Thymic Architecture of Motheaten ($Hcph^{me}/Hcph^{me}$) Mice

The thymuses of $Hcph^{me}/Hcph^{me}$ mice were smaller than the aged-matched controls, the moderate involution including both the cortex and medulla. Staining of

TABLE II Comparison of Thymic Microenvironment on Experimented Strains

| mAb and changes | | BALB/c | C57BL/6 | +/*Hcph*$^{me}$ | +/*aly* | NZB | SM/J | NOD *scid* | NOD-/*Hcph*$^{me}$ | *Hcph*$^{me}$ | *aly*/*aly* |
|---|---|---|---|---|---|---|---|---|---|---|---|
| Thymic Structure | | | | | | | | | | | |
| | Involution of the thymus | − | − | − | − | − | − | − | +++ | ++ | − |
| | Atrophy of the lobules | − | − | − | − | − | − | − | − | − | − |
| | Increase of the cysts | − | − | − | − | − | − | − | − | − | +++ |
| Thymic Epithelium | | | | | | | | | | | |
| Cortex | Atrophy of the cortex (MTS1, 44) | − | − | − | − | − | − | − | + | + | − |
| | Isolated clusters in cortex (MTS10) | − | − | − | − | ++ | + | + | − | − | +++ |
| | Diffused and irregular networks (MTS1, 5, 44) | − | − | − | − | +++ | ++ | + | ++ | − | +++ |
| | Increased epithelial cell free regions (MTS1, 5, 44) | − | − | − | − | +++ | + | + | + | − | +++ |
| | Small meshworks (MTS1, 5, 44) | − | − | − | − | − | − | − | +++ | ++ | − |
| Medulla | Atrophy of the medulla (MTS1, 10) | − | − | − | − | − | − | − | + | + | − |
| | Irregular-shaped medulla (MTS1, 10) | − | − | − | − | +++ | ++ | + | +++ | − | +++ |
| | Decrease positive cells in the medulla (MTS1, 10, 33) | − | − | − | − | ++ | + | + | +++ | − | +++ |
| | Decrease isolated epithelium in the medulla (MTS20, 24) | − | − | − | − | +++ | − | ++ | ++ | − | +++ |
| Cortico medullary junction | Unclear corticomedullary junction (hematoxylin and eosin staining) | − | − | − | − | + | − | + | +++ | − | +++ |
| Thymocytes | | | | | | | | | | | |
| | Increased staining of TSA-1 (MTS35) | − | − | − | − | − | ++ | − | −~+ | ++ | + |
| Nonepithelial Stromal Cells | | | | | | | | | | | |
| Extracellular matrix | Increased MTS16 expression | − | − | − | − | − | − | − | +++ | − | +++ |
| | Decreased MTS16 expression | − | − | − | − | + | − | + | − | − | − |

Abnormalities were assessed by the following grading system: +++: severe abnormalities; ++: moderate abnormalities; +: mild abnormalities; −: no abnormalities detected.

the epithelial networks (MTS44- and MTS10-positive, respectively), however, was similar to the control strains (Figs. 2 and 3). *Hcph*$^{me}$/*Hcph*$^{me}$ mice also demonstrated elevated MTS35-positive thymocytes in the corticomedullary and medullary regions (Fig. 4). Staining with MTS16 (Fig. 5), MTS12, Mac-1, or MTS28 was normal as compared with + /*Hcph*$^{me}$-heterozygous, C57BL/6, or BALB/c mice.

### Thymic Architecture of *aly/aly* Mice

Mice homozygous for the alymphoid (*aly*) mutations (*aly/aly*) exhibited marked alterations in their thymic architecture. The most dramatic and distinguishing feature was the presence of epithelial cysts identified by hematoxylin and eosin staining, MTS1, or anti-keratin antibody; these were at the corticomedullary junction or medulla. The cortical epithelial network was severely disrupted and irregular when stained with MTS1, MTS5, or MTS44 (Fig. 2), with many areas actually lacking MTS44-positive epithelial cells. In addition, isolated clusters of MTS10 staining were found in the cortex. The medullary epithelium or *aly/aly* mice was also severely disrupted, as shown by staining with MTS1 or MTS10 (Fig. 3), with fewer of the clusters positive for MTS33, MTS20, or MTS24 (Fig. 3). The *aly/aly* mice had elevated MTS35-positive thymocytes around the corticomedullary junction (Fig. 4), whereas they are normally in the outer cortex. However, staining of thymocytes with MTS33 or MTS37 was similar to + /*aly*-heterozygous, C57BL/6, or BALB/c mice. There was marked increase in expression of the extracellular matrix recognized by MTS16, the staining being more aggregated and irregularly shaped. Thymic staining with MTS12, Mac-1, or MTS28 was essentially normal.

### DISCUSSION

One of the striking features of the thymus is the phylogenetically conserved cellular content and structural organization of the stromal components. These constitute the specific microenvironment of this organ and orchestrate the induction and regulation of thymopoiesis in such a manner that pathological states rarely arise. The corollary of this is that defects in the microenvironment at the structural and/or cellular levels could predispose the individual to T-cell-based disorders such as autoimmunity or immunodeficiency. In this regard, we have previously shown that the lupus-prone NZB mice and related strains are characterized by abnormalities in the thymic microenvironment manifest as ectopic expression of medullary epithelial antigens in the cortex and the marked expansion of lymphocyte-rich areas lacking epithelial cells. The abnormalities have been already occurring since 1 month before disease and become worse with aging (Watanabe et al., 1993). The present study was undertaken to determine if these thymic microenvironment abnormalities are generic to other autoimmune and also immunodeficient mice.

Collectively, the data generated in this study are consistent with defects in thymic architecture in several of the murine strains evaluated. Because these are manifest before overt signs of disease, they do not develop as a consequence of disease and may thus be of etiologic significance. The changes were specific for the disease-prone mice and were never observed in a panel of normal mice strains (e.g., BALB/c, CBA, and C57BL/6). SM/J (thymotoxic autoantibodies) and NOD mice had extensive epithelial-cell-free regions and disrupted cortical epithelial networks similar to those of NZB and other models of murine lupus (Watanabe et al., 1993; Takeoka et al., 1995a; 1995b). These were also observed in one of the immunodeficient models, *aly/aly* mice. However, the thymic abnormalities of NOD-*scid* mice—severe thymic involution, poorly defined cortex, and rudimentary medulla increased extracellular matrix are more typical of aged mice such as (Takeoka et al., 1996) than murine lupus. These changes are unlikely to be related to any disease processes, but rather a consequence of the lack of T cells, particularly mature T cells, which are required for epithelial-cell maturation and expansion. The thymus in motheaten (*Hpch*$^{me}$/*Hpch*$^{me}$) mice also showed some atrophy, but the stromal architecture was relatively normal.

Given the importance of cortical epithelium in thymocyte differentiation and positive and negative selection (Berg et al., 1989; Ashton-Rickardt et al., 1993; Boyd et al., 1993), any abnormalities in this region in particular are very likely to be translated into defective T-cell development and hence predisposition to disease. The role of the medullary epithelium is less clear, but it presumably has a role in the final maturation of thymocytes, including their capacity to migrate to the periphery; it may also induce tolerance through energy. A possibility in many of the models (e.g., NZB, SM/J, and NOD), therefore, is that the presence of medullary epithelium in the cortex delivers premature maturation signals to surrounding lymphocytes, expediting their progression to functionally competent cells, thereby escaping normal tolerance induction mechanisms. Because these alterations were not found in *Hpch*$^{me}$/*Hpch*$^{me}$, they are generic to all autoimmunity-prone mice.

The significance of the ECF is still unclear. By definition, they lack epithelial cells, but they have a minor population of supporting stromal cells and are filled with thymocytes of multiple phenotypes, including dividing cells (Boyd et al., unpublished observations). Our working hypothesis is that these thymocytes accumulate through lack of normal deletion mechanisms, creating space through the elasticity of the stromal architecture. They could, therefore, harbor potentially autoreactive cells.

The extracellular matrix, which is composed of a mixture of collagen, reticulin fibers, glycosaminoglycans, and glycoproteins surrounding the epithelial cells, is also important to thymic integrity and thymocyte development, including mediating cell migration and binding of soluble cytokines (Lannes-Vieira et al., 1991; Boyd et al., 1993; Savino et al., 1993). It has been reported that 12-month-old normal mice exhibit a denser and irregular-shaped extracellular matrix pattern than young adult normal mice (Lannes-Vieira et al., 1991; Takeoka et al., in press). Our data demonstrate that NOD-*scid* or *aly*/*aly* mice exhibited increased number and density of extracellular matrix. Further, the extracellular matrix was aggregated and expressed a large irregular shape in the thymus of *aly*/*aly* mice. The abnormalities of the extracellular matrix in thymus of NOD-*scid* or *aly*/*aly* mice were similar to age-related degenerative changes in normal mice, and may be a factor in immunosenescence due to involution of thymus. On the other hand, the extracellular matrix was decreased in NZB and NOD mice as compared to C57BL/6, BALB/c, SM/J, or heterozygous controls. This may again disrupt T normal thymopoiesis through reduced cell migration and binding of soluble cytokines.

SM/J mice possess some thymic epithelial abnormalities common to murine SLE, but one important difference was found by staining with MTS35. This mAb recognizes the thymic shared antigen-1 (TSA-1), a marker of immature thymocytes having an inverse relationship with CD3 expression (Randle et al., 1993), and isolated thymic stromal cells, which include dendritic cells and a medullary epithelial-cell subset. It is, however, upregulated on activated mature T cells (Kosugi et al., 1994). This accumulation of TSA-1-positive thymocytes may be related to abnormalities in the thymic epithelium and could either be due to disruption of the cortical network, allowing release of immature thymocytes, or activation of a select subset in the medulla. In fact, SM/J mice have a high level of naturally occurring thymocytotxic autoantibody (NTA) that may contribute to either of these possibilities (Mittal et al., 1970). Motheaten (*Hpch*$^{me}$/*Hpch*$^{me}$) mice did not have marked abnormalities in the stroma, but did have the elevated MTS35-positive cells at the corticomedullary junction. The relationship between this and the high levels of IgM, which are 25 to 50 times greater than that of control mice (Shultz and Green, 1976), is unknown.

Collectively, these data clearly demonstrate that pathological states such as autoimmunity and immunodeficiency are associated with marked abnormalities in the thymic microenvironment. Although a direct causal link between these two has yet to be verified, this study underlies the importance of understanding the nature and function of the thymic microenvironment as a prelude to determining the etiology of several T-cell-based disorders. We are currently pursuing the identification and gene cloning of the antigens investigated in this study.

### MATERIAL AND METHODS

### Mice

Female NZB/BlnJ, C57BL/6J, BALB/cJ, SM/J, or NOD/Ltz-+ /+ mice, and male NOD/Ltz-*scid*/*sz*

(herein referred to as NZB, C57BL/6, BALB/c, SM/J, NOD, or NOD-*scid*) mice were obtained from Jackson Laboratory (Bar Harbor, ME), and were maintained by the Animal Resource Services of the University of California at Davis. Female or male motheaten C57BL/6J-$Hcph^{me}/Hcph^{me}$ and heterozygous control C57BL/6J-+ /$Hcph^{me}$ (herein referred to as $Hcph^{me}/Hcph^{me}$ and + /$Hcph^{me}$-heterozygous) mice were maintained by Jackson Laboratory. Female or male ALY/Nsc Jcl-*aly*/*aly* mice and heterozygous control ALY/Nsc Jcl-+ /*aly* mice (herein referred to as *aly*/*aly* and + /*aly*-heterozygous) were obtained from CLEA Japan (Osaka), and were maintained by the Institute of Bio-Active Science, Nippon Zoki Pharmaceutical (Hyogo). Groups of up to ten mice were individually studied, and 4–10-week-old mice were used. Mice of both sexes were studied. Because no sex differences were observed, the data from males and females mice were combined.

### Monoclonal Antibodies (mAbs)

A panel of 14 mAb with previously defined specificity for mouse thymic stromal (MTS) elements and thymocytes were used in this study. The MTS mAbs were prepared from the fusion of P3-NS-1-Ag4-1 (NS-1) cells with spleen cells or popliteal lymph node cells from LOU/M rats immunized with enriched mouse thymic stromal-cell suspensions (Godfrey et al., 1990). The characteristics of each of these mAb were described before, summarized in Table I, and illustrated in Fig. 1.

### Histochemistry

Thymi were removed from mice, snap-frozen in dry, ice-cold 2-methyl butane, or dry, ice-cold ethanol, and embedded in TISSUE-TEC\T (Miles Laboratories, Naperville, IN). Freshly cut sections (5 μm) were mounted on clean glass slides coated with poly-L-lysine (Sigma Chemical, St. Louis), and rapidly air dried. They were stored at − 80°C until used for immunohistochemical staining. Sections were fixed with acetone at − 20°C for 5 min before staining.

Fixed sections were stained by hematoxylin and eosin staining. For immunohistochemistry, the sections were incubated for 30 min at room temperature with normal goat serum diluted 1lmX5 in 0.05 M Tris-buffered saline (TBS), pH 7.6, to block nonspecific staining of secondary antibody. Sections were then stained using an avidin-biotin technique based on the system of Wood and Warnke (1981). Sections were incubated with the appropriate and optimal dilution of MTS mAbs or other primary antibodies for 1 h at room temperature in a moist chamber, and then washed three times in TBS for 5 min with gentle shaking. They were then incubated with biotinylated goat anti-rat Ig (Tago, Burlingame, CA) diluted 1lmX200 in TBS for 30 min at room temperature and washed with TBS. Biotinylated goat anti-rabbit Ig (Tago) was used with anti-keratin at a 1lmX200 dilution in TBS. The alkaline phosphatase-conjugated streptavidin-biotin complex (AB complex/AP) (Dako, Carpinteria, CA) was freshly prepared and applied to sections for 30 min. After washing in TBS, the substrate (Vector Red; Vector, Burlingame, CA) in 0.1 M TBS, pH 8.2, was applied to the sections with levamisole to block endogenous alkaline phosphatase activity. After washing in TBS, they were mounted with glycerol in phosphate-buffered saline (mounting medium; Sigma Diagnostics, St. Louis) and cover slipped.

The slides were viewed using an Olympus BH-2 with a Bio-Rad MRC 600 laser confocal microscope (Bio-Rad Laboratories, Hercules, CA), a GHS filter block, and a #2 neutral density filter. The resulting confocal images were analyzed using the SOM program or GEOG.LOU program for comparison of the intensity integrated with the Bio-Rad confocal system.

### *References*

Ashton-Rickardt, P. G., Van Kaer, L., Schumacher, T. N. M., Ploegh, H. L., and Tonegawa, S. (1993). Peptide contributes to the specificity of positive selection of $CD8^+$ T cells in the thymus. *Cell* **73**: 1041–1049.

Berg, L. J., Pullen, A. M., Fazekas de St. Groth, B., Mathis, D., Benoist, C., and Davis, M. M. (1989). Antigen/MHC-specific T cells are preferentially exported from the thymus in the presence of their MHC ligand. *Cell* **58**: 1035–1046.

Blackburn, C. C., Augustine, C. L., Harvey, R. P., Malin, M. A., Boyd, R. L., Miller, J. F. A. P., and Morahan, G. (1996). The *nu* gene acts cell-autonomously and is required for differentiation of thymic epithelial progenitors. *Proc. Natl. Acad. Sci. USA* **93**: 5742–5746.

Boyd, R. L., Tucek, C. L., Godfrey, D. I., Izon, D. J., Wilson, T. J., Davidson, N. J., Bean, A. G. D., Ladyman, H. M., Ritter, M. A., and Hugo, P. (1993). The thymic microenvironment. *Immunol. Today* **14**: 445–459.

Boyd, R. L., Wilson, T. J., Bean, A. G., Ward, H. A., Gershwin, M. E. (1992). Phenotypic characterization of chicken thymic stromal elements. *Dev. Immunol.* **2**: 51–66.

Eisenberg, R. A., Theofilopoulos, A. N., Andrews, B. S., Peters, C. J., Thor, L., and Dixon, F. J. (1979). Natural thymocytotoxic autoantibodies in autoimmune and normal mice. *J. Immunol.* **122**: 2272–2278.

Godfrey, D. I., Izon, D. J., Tucek, C. L., Wilson, T. J., and Boyd, R. L. (1990). The phenotypic heterogeneity of mouse thymic stromal cells. *Immunology* **70**: 66–74.

Godfrey, D. I., Masciantonio, M., Tucek, C. L., Malin, M., Boyd, R. L., and Hugo, P. (1992). Thymic shared antigen-1. A novel thymocyte marker discriminating immature from mature thymocyte subsets. *J. Immunol.* **148**: 2006–2011.

Goldblum, R., Pillarisetty, R., and Talal, N. (1975). Independent appearance of anti-thymocyte and anti-RNA antibodies in NZB/NZW F1 mice. *Immunology* **28**: 621–628.

Green, M. C., and Shultz, L. D. (1975). Motheaten, an immunodeficient mutant of the mouse. I. Genetics and pathology. *J. Hered.* **66**: 250–258.

Huang, S.-W., Lattos, D. B., Nelson, D. B., Reeb, K., and Hong, R. (1973). Antibody-associated lymphotoxin in acute infection. *J. Clin. Invest* **52**: 1033–1040.

Kampinga, J., Berges, S., Boyd, R. L., Brekelmans, P., Colic, M., van Ewijk, W., Kendall, M., Ladyman, H., Nieuwenhuis, P., Ritter, M., Schuurman, H.-J., and Tournefier, A. (1989). Thymic epithelial antibodies: Immunohistological analysis and introduction of CTES nomenclature. *Thymus* **13**: 165–173.

Kosugi, A., Saitoh, S., Narumiya, S., Miyake, K., and Hamaoka, T. (1994). Activation-induced expression of thymic shared antigen-1 on T lymphocytes and its inhibitory role for TCR-mediated IL-2 production. *Int. Immunol.* **6**: 1967–1976.

Lannes-Vieira, J., Dardenne, M., and Savino, W. (1991). Extracellular matrix components of the mouse thymus microenvironment: Ontogenetic studies and modulation by glucocorticoid hormones. *J. Histochem. Cytochem.* **39**: 1539–1546.

Mittal, K. K., Rossen, R. D., Sharp, J. T., Lidsky, M. D., and Butler, W. T. (1970). Lymphocyte cytotoxic antibodies in systemic lupus erythematosus. *Nature* **225**: 1255–1256.

Miyawaki, S., Nakamura, Y., Suzuka, H., Koba, M., Yasumizu, R., Ikehara, S., and Shibata, Y. (1994). A new mutation, aly, that induces a generalized lack of lymph nodes accompanied by immunodeficiency in mice. *Eur. J. Immunol.* **24**: 429–434.

Oukka, M., Cohen-Tannoudji, M., Tanaka, Y., Babinet, C., and Kosmatopoulos, K. (1996). Medullary thymic epithelial cells induce tolerance to intracellular proteins. *J. Immunol.* **156**: 968–975.

Randle, E. S., Wanders, G. A., Masciantonio, M., Godfrey, D. I., and Boyd, R. L. (1993). A lymphostromal molecule, Thymic Shared Antigen-1, regulates early thymocyte development in fetal thymus organ culture. *J. Immunol.* **151**: 6027–6035.

Ritter, M. A., and Boyd, R. L. (1993). Development in the thymus: It takes two to tango. *Immunol. Today* **14**: 462–469.

Savino, W., Villa-Verde, D. M., and Lannes-Vieira, J. (1993). Extracellular matrix proteins in intrathymic T-cell migration and differentiation? *Immunol. Today* **14**: 158–161.

Schönrich, G., Momburg, F., Hämmerling, G. J., and Arnold, B. (1992). Energy induced by thymic medullary epithelium. *Eur. J. Immunol.* **22**: 1687–1691.

Shirai, T., and Mellors, R. C. (1971). Natural thymocytotoxic autoantibody and reactive antigen in New Zealand Black and other mice. *Proc. Natl. Acad. Sci. USA* **68**: 1412–1415.

Shultz, L. D., and Green, M. C. (1976). Motheaten, an immunodeficient mutant of the mice. II. Depressed immune competence and elevated serum immunogloblins. *J. Immunol.* **116**: 936–943.

Shultz, L. D., Schweitzer, P. A., Rajan, T. V., Yi, T., Ihle, J. N., Matthews, R. J., Thomas, M. L., and Beier, D. R. (1993). Mutations at the murine motheaten locus are within the hematopoietic cell protein-tyrosine phosphatase (*Hcph*) gene. *Cell* **73**: 1445–1454.

Takeoka, Y., Chen, S.-Y., Yago, H., Boyd, R. L., Suehiro, S., Shultz, L. D., Ansari, A. A., and Gershwin, M. E. (1996). The murine thymic microenvironment: Changes with age. *Int. Arch. Allergy Immunol.* **111**: 5–12.

Takeoka, Y., Whitmer, K. J., Chen, S.-Y., Ansari, A. A., Boyd, R. L., Schultz, L. D., Suehiro, S., and Gershwin, M. E. (1995b). Thymic epithelial cell abnormalities in (NZB × $H\text{-}2^u$) F1 mice. *Clin. Immunol. Immunopathol.* **76**: 297–307.

Takeoka, Y., Yoshida, S. H., Van de Water, J., Boyd, R. L., Suehiro, S., Ansari, A. A., and Gershwin, M. E. (1995a). Thymic microenvironmental abnormalities in MRL/MP-*lpr*/*lpr*, BXSB/MpJ*Yaa* and C3H/HeJ-*gld*/*gld* mice. *J. Autoimmunity* **8**: 145–161.

Terasaki, P. I., Mottironi, V. D., and Barnett, E. V. (1970). Cytotoxins in disease. Autocytotoxins in lupus. *New Engl. J. Med.* **283**: 724–748.

Tochino, Y. (1987). The NOD mouse as a model of type I diabetes. *Crit. Rev. Immunol.* **8**: 49–81.

Van de Wijngert, F. P., Rademakers, L. H., Schuurman, H. J., de Weger, R. A., and Kater, L. (1983). Identification and in situ localization of the "thymic nurse cell" in man. *J. Immunol.* **130**: 2348–2351.

Watanabe, Y., Naiki, M., Wilson, T., Godfrey, D., Chiang, B.-L., Boyd, R., Ansari, A., and Gershwin, M. E. (1993). Thymic microenvironmental abnormalities and thymic selection in NZB.H-$2^{bm12}$ mice. *J. Immunol.* **150**: 4702–4712.

Wood, G. S., and Warnke, R. (1981). Suppression of endogenous avidin-binding activity in tissue and it relevance to biotin-avidin detection systems. *J. Histochem. Cytochem.* **29**: 1196–1204.

